# Supplementary figures and images for: Salivary surprise: Symmerista caterpillars anoint petioles with red saliva after clipping leaves
Source: PLoS One. 2022 Mar 16;17(3):e0265490. doi: 10.1371/journal.pone.0265490 (PMC8926259; doi:10.1371/journal.pone.0265490)

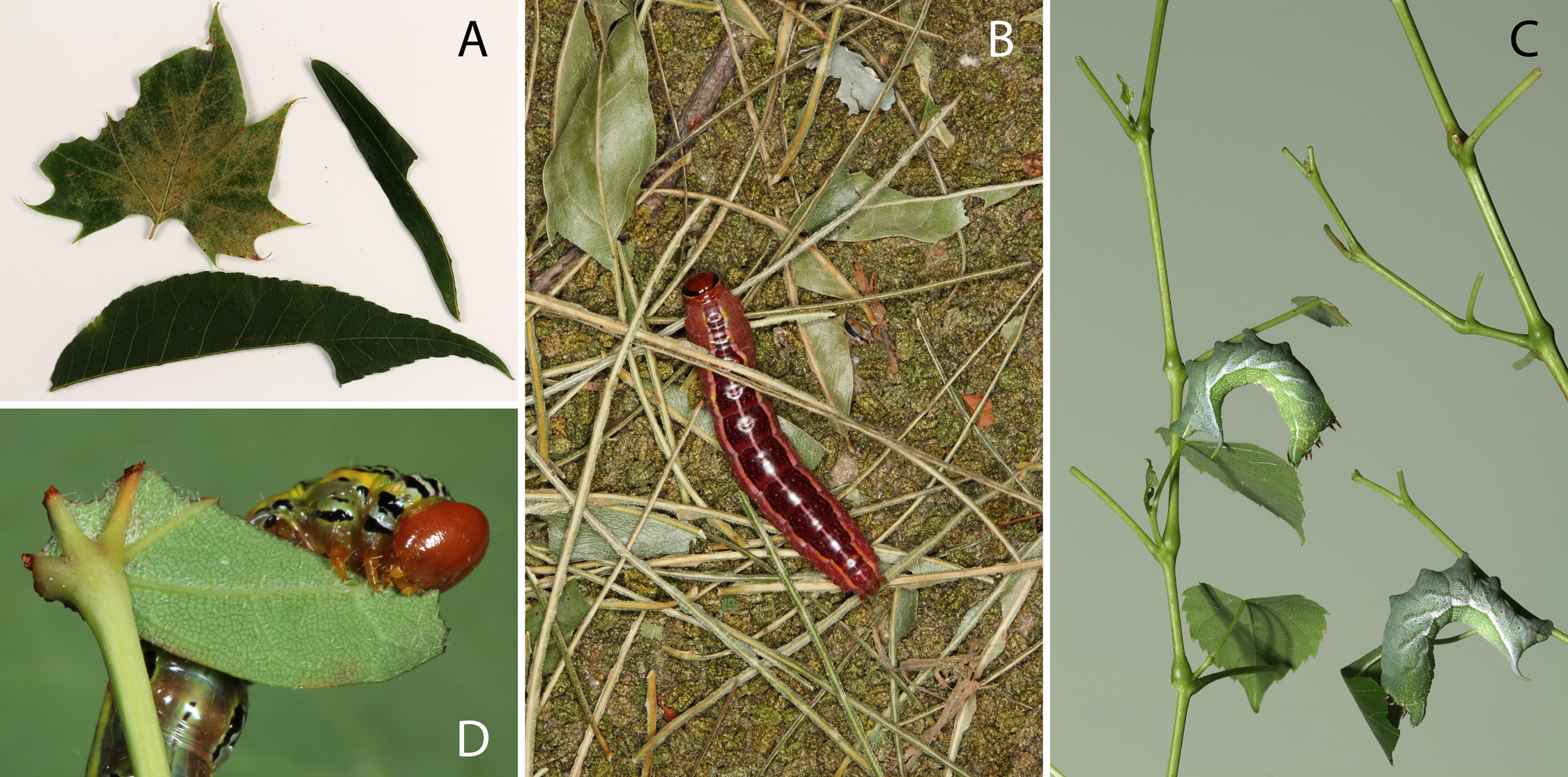

Supplement: S1 Fig — (A) Clipped leaves of sycamore (Platanus occidentalis, Platanaceae) and willow oak (Quercus phellos, Fagaceae) and clipped leaflet of pecan (Carya illinoinensis leaf, Juglandaceae) found on the ground in Conway, Arkansas. (B) Larva of Lochmaeus manteo (Notodontidae) dispersing from a willow oak tree (Q. phellos) to pupate. The ground is covered with clipped oak midribs and caterpillar frass due to an L. manteo outbreak June 2013 in Conway, Arkansas. (C) Two larvae of Darapsa myron (Sphingidae) on excised stems of the vine Ampelopsis cordata (Vitaceae). The larvae ate entire leaf blades, then sequentially consumed portions of the petiole and rubbed saliva on the petiole stubs after finishing each segment. The larvae never clipped the petioles despite the similar appearance of the A. cordata petiole stubs to petioles clipped by other caterpillar species (Figs 1–3 and S4). (D) Final instar of Symmerista leucitys feeding on sugar maple, Acer saccharum (Sapindaceae). The larva did not clip the petiole, but instead applied red saliva to each major leaf vein after consuming a portion of the leaf. (TIF) [file pone.0265490.s001.tif]

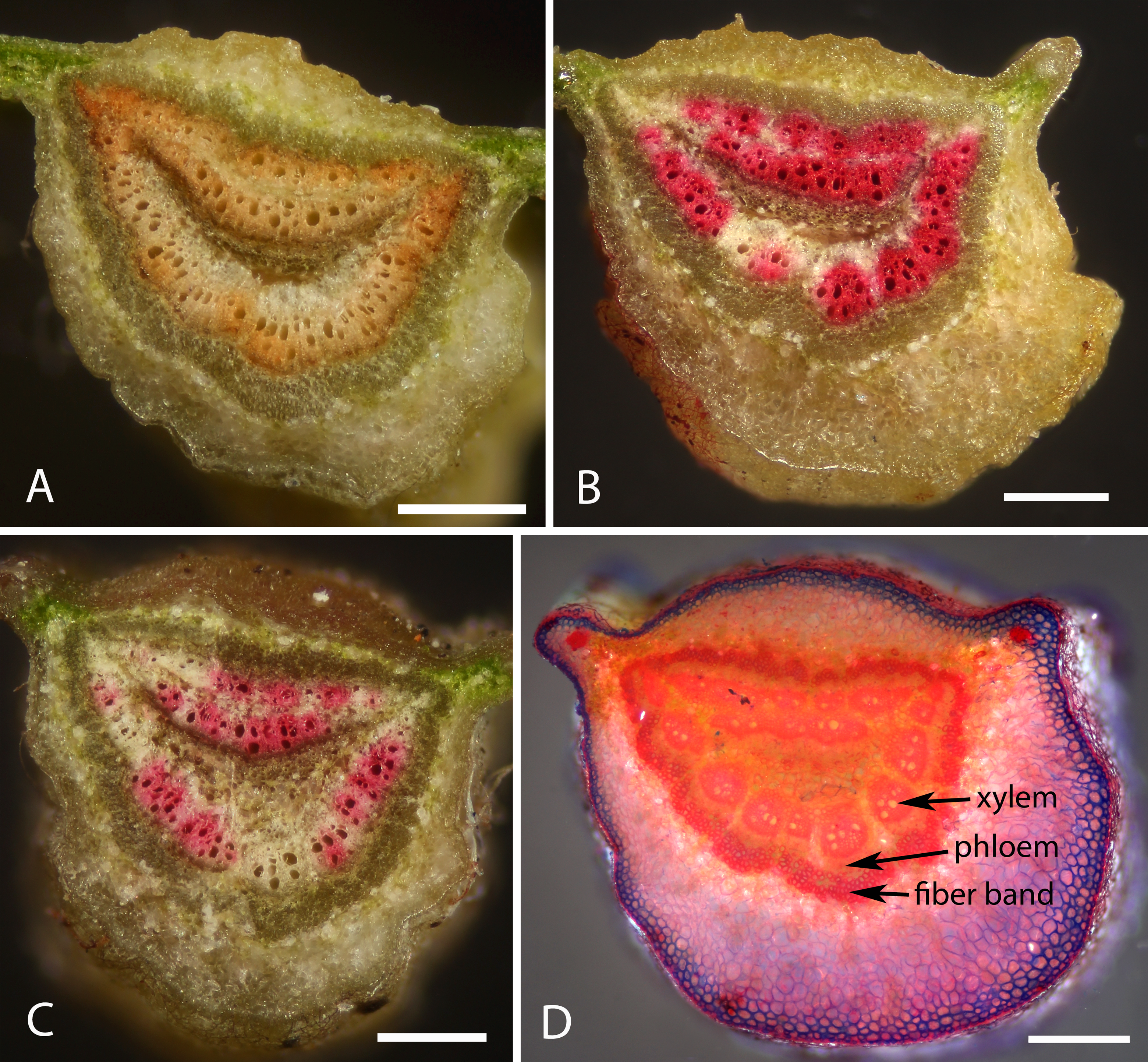

Supplement: S2 Fig — (A) Petiole that was clipped by a final instar of S. albifrons, then severed with a razor blade five minutes later 2.5mm from the petiole tip. The red salivary pigment moved into the petiole staining the xylem. (B) Petiole xylem stained by safranin O dye (0.2%) pulled up the xylem by transpiration. (C) Petiole stub treated with 1μl safranin O dye (0.2%). After 5 minutes, the petiole was severed 2.5mm from the initial cut. The dye moved in a retrograde direction down the xylem staining the same tissues as S. albifrons pigment in (A) and dye in (B). The petiole slices in A-C were photographed dry to prevent pigment and dye from dissolving or spreading to adjacent tissues; some distortion of the exterior surface of the slices resulted. (D) Thin slice of petiole stained with astra blue followed by safranin O to illustrate the position of vascular tissues. The phloem is located between the xylem and fiber band (sclerenchyma) (AL Filartiga pers. comm.). Scale bars equal 0.3 mm. (TIF) [file pone.0265490.s002.tif]

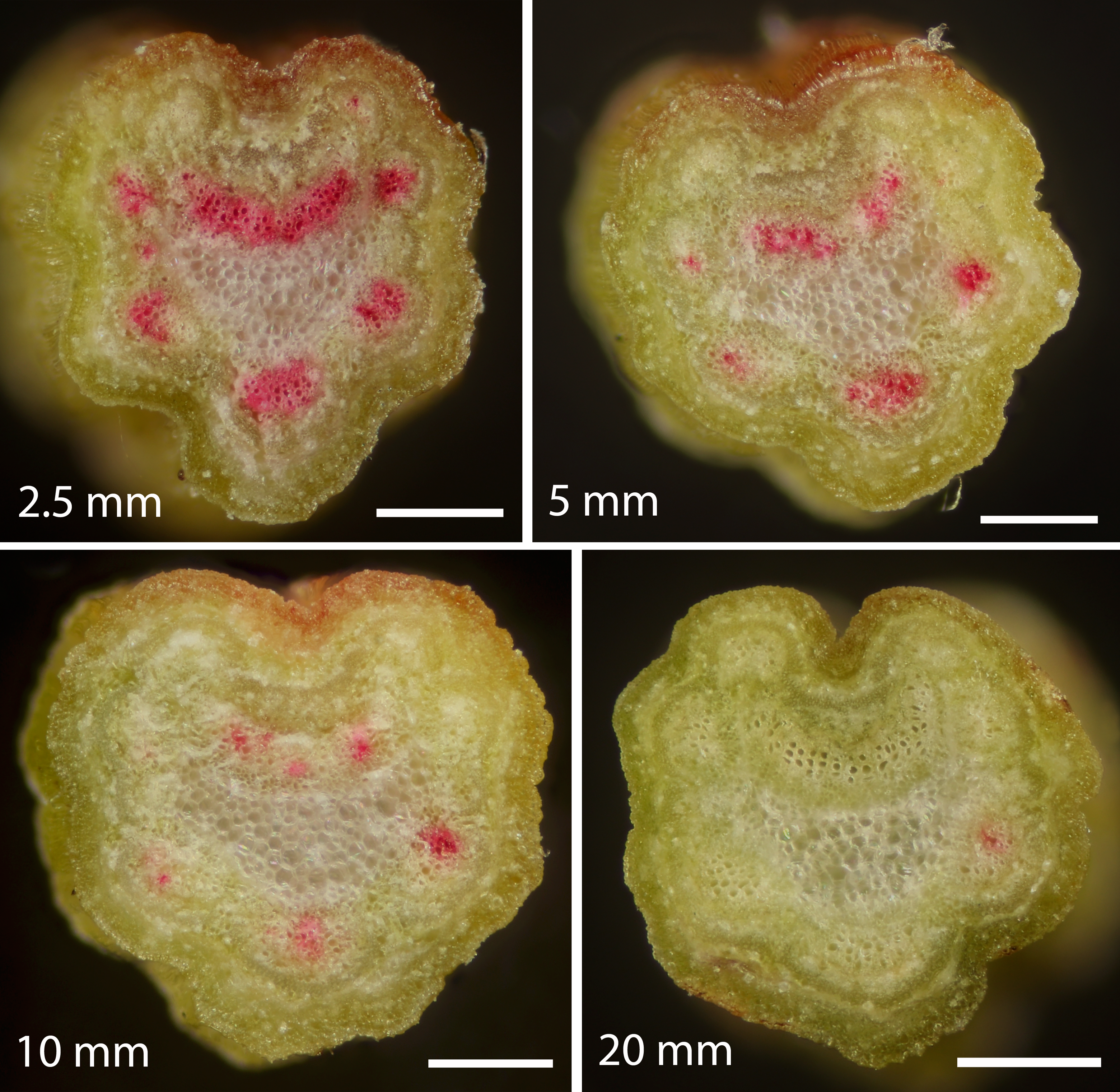

Supplement: S3 Fig — The petiole was severed, then 1μl safranin O dye (0.2%) was placed on the petiole stub. After 5 minutes, cross sections of the petiole were cut 2.5, 5, 10 and 20 mm down the petiole. The dye moved in some xylem vessels over 20 mm in five minutes. Scale bars equal 0.3 mm. (TIF) [file pone.0265490.s003.tif]

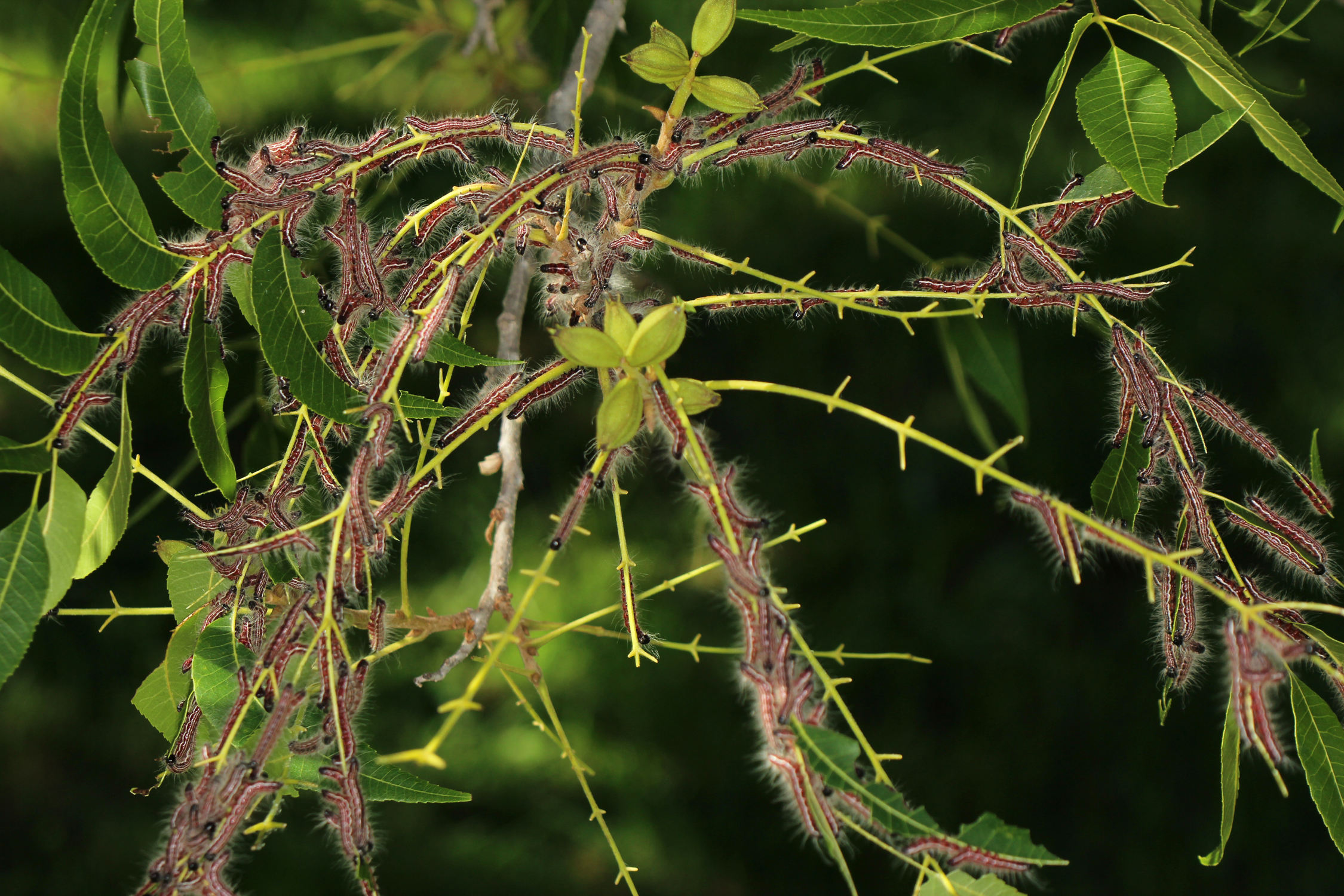

Supplement: S4 Fig — The larvae consumed entire leaflets except for the midribs, then repeatedly clipped each midrib leaving only the rachis and short stubs. (TIF) [file pone.0265490.s004.tif]
